# Catching’s Compendium of Practical Dentistry for 1892

**Published:** 1893-04

**Authors:** 


### Bibliography.

---

Catching's Compendium of Practical Dentistry for 1892. By B. H. Catching, D.D.S., Editor and Publisher, Atlanta, Ga.

The dental profession is again under obligations to the faithful compiler of this valuable addition to its standard literature. It appears for 1892 with improvements in many directions. This was to be expected, as such a work requires special training; and as no standard existed as a guide, the editor is to be congratulated that he has been able to make it a necessity for those unable or unwilling to gather ideas from a mass of material, and, as it were, embalm them for future reference.

To the superficial reader this may seem a task readily accomplished, simply requiring acute observation and careful culling. When, however, it is understood that the condensation means not only care in reading, but a power of apprehending the idea of the writer, it assumes an altogether different character. That Dr. Catching has succeeded in this is abundantly proven upon the well-filled pages. It is not his fault that the matter does not seem to show any great advance in dental work.

When the large amount of printed matter is laid before professional readers monthly, the natural feeling is that marked progress is being made in every direction, but the skilled dissector of ideas lays a ruthless hand on this opinion and exhibits comparatively meagre results for the year, and forces the question, Are we making the scientific progress we should?

Not the least valuable part of this compendium is that it represents us as we are, and while the exhibition may not increase self-satisfaction with our work, it may, it is hoped, lead to increased activity and thoroughness.

A compendium of dentistry, it seems to the writer, should have a broader scope than has been given to this. Dentistry to-day is not confined to the English-speaking races, nor do they have a monopoly of the best work. It has the entire civilized world for its field of labor.

It, therefore, would seem appropriate—even more than that, necessary—to glean from all parts in order to make such a work a true compendium of "practical dentistry." If the scientific side be added to this, as it should be, it will be still more difficult to confine it to any one country.

The difficulties in accomplishing this are many and may be insurmountable, but if the entire field of dental periodical literature could be treated as carefully as the work has been done for this country, it would add immensely to succeeding volumes. As it is, it lacks just this to make it of interest to the scientific worker.

While it has its limitation as an exponent of the dental thought of the year, it remains invaluable as a work of reference within the lines the compiler has laid out for himself.

The general make-up is very satisfactory and reflects credit on all concerned in its publication.

